# Followers' Choice: The Trends Transforming Precision Medicine, Synthetic Biology, and Sustainable Microbiology

**DOI:** 10.1111/1751-7915.70370

**Published:** 2026-05-05

**Authors:** Patricia Bernal, Rocío Palacios‐Ferrer, Juan L. Ramos

**Affiliations:** ^1^ Departmento de Microbiología, Facultad de Biología Universidad de Sevilla Seville Spain; ^2^ Estación Experimental del Zaidín CSIC Granada Spain; ^3^ Programa de Doctorado en Bioquímica y Biología Molecular University of Granada Granada Spain

The diffusion of science is evolving rapidly, with social networks playing an increasingly essential role. By analysing the journal's accounts on X (@MicrobialBiote1) and BlueSky (@microbiotech.bsky.social), we identified recent microbiology and biotechnology studies that have captured the interest and engagement of our followers, reflecting the topics that resonate most with our scientific community. Together, these articles reveal emerging trends in precision medicine, microbial ecology, synthetic biology, and sustainable biotechnology.

A central priority in the broader effort to combat diseases is the development of targeted microbial therapies. Choudhury et al. (https://doi.org/10.1111/1751‐7915.70241) present an innovative precision antimicrobial strategy against *Fusobacterium nucleatum* , a bacterium associated with colorectal cancer. By engineering *Lactococcus lactis* to deliver guided antimicrobial peptides (gAMPs), these researchers achieved selective inhibition of the pathogen while preserving the beneficial microbiota. The system demonstrated strong specificity, reduced toxicity, and the ability to maintain microbial diversity in simulated gut environments, emphasizing the potential of engineered probiotics as next‐generation therapeutics.

The therapeutic potential of probiotics is further illustrated in a study by Wang et al. on chronic spontaneous urticaria, in which *Lactobacillus paragasseri* LG‐1 was shown to modulate metabolism, restore microbiota balance, and reduce inflammation through immune pathway regulation (https://doi.org/10.1111/1751‐7915.70316). The interest in this area is also reflected in advances in probiotic microencapsulation, which aim to enhance the stability and targeted delivery of beneficial microbes, paving the way for more effective and personalized therapeutic applications (Zhu et al., https://doi.org/10.1111/1751‐7915.70305).

Expanding beyond human health, another study by Vidal et al. (https://doi.org/10.1111/1751‐7915.70286) explores how Earth's subsurface microbiome can inform the search for extraterrestrial life. Microorganisms thriving in extreme, low‐energy environments beneath the Earth's surface provide valuable analogues for potential habitats on Mars and icy moons such as Europa and Enceladus. Their findings suggest that extraterrestrial life may be slow‐growing and difficult to detect, reinforcing the need for advanced detection technologies and refined biosignature identification.

Sustainability in agriculture has emerged as a crucial approach to ensuring that food production is balanced with the protection of environmental resources. Microbial volatile organic compounds (VOCs) are highlighted as promising alternatives to chemical pesticides, capable of inhibiting pathogens and promoting plant health (Belt et al., https://doi.org/10.1111/1751‐7915.70313). However, translating laboratory findings into field applications remains challenging due to environmental variability and technical limitations in VOC detection. Future progress will depend on integrating ecological complexity into experimental design. In parallel, the group of Brajesh Singh has made relevant contributions to this rapidly expanding field as reviewed in (Xiong et al., https://doi.org/10.1038/s41579‐026‐01290‐2).

At the cellular level, advances in bacterial organization and regulation were also featured. Research on bacterial microcompartments (BMCs) in *Salmonella* demonstrates how engineered hybrid organelles can reorganize metabolic pathways, offering insights into microbial efficiency and synthetic biology applications (Chang et al., https://doi.org/10.1111/1751‐7915.70301). Complementing this, another study by Fernández‐Fernández et al. (https://doi.org/10.1111/1751‐7915.70312) reveals how variability in promoter regions of epigenetically regulated operons enables bacteria to fine‐tune gene expression and adapt rapidly to environmental pressures. Deciphering these molecular mechanisms is critical for advancing innovative strategies to combat antibiotic resistance, an escalating public health threat expected to become the leading global cause of death worldwide by 2050 (Brüssow, https://doi.org/10.1111/1751‐7915.14510).

Industrial biotechnology innovations are likewise highly engaged on social networks. Matamouros et al. describe a high‐throughput platform for signal peptide screening in *Corynebacterium glutamicum*, enabling more efficient secretion of recombinant proteins, reducing development time and costs (Matamouros et al., https://doi.org/10.1111/1751‐7915.70299).

Finally, sustainability is addressed through the development of hybrid microbiomes for bioplastic production by Zini et al. (https://doi.org/10.1111/1751‐7915.70302). These authors integrated engineered cyanobacteria into natural microbial communities and created a robust system capable of producing biodegradable plastics under scalable, non‐sterile conditions.

Collectively, these follower‐selected studies underscore the growing importance of microbial innovation across medicine, agriculture, energy, and industry, highlighting how advances in microbiology continue to shape solutions to some of today's most pressing global challenges.

## Author Contributions

**Patricia Bernal:** writing – original draft, writing – review and editing, data curation. **Rocío Palacios‐Ferrer:** writing – original draft, writing – review and editing, data curation. **Juan L. Ramos:** conceptualization, writing – original draft, writing – review and editing, supervision.

## Funding

The authors have nothing to report.

## Conflicts of Interest

The authors declared no conflicts of interest.

## Data Availability

Data sharing not applicable to this article as no datasets were generated or analysed during the current study.

